# Topography of violent intraspecific aggression in a subset of dogs from directionally selected lines of *Canis familiaris*

**DOI:** 10.1371/journal.pone.0335278

**Published:** 2025-11-19

**Authors:** Victoria A. Cussen, Qinyi Lu, Pamela J. Reid

**Affiliations:** 1 Behavioral Sciences Team, The American Society for the Prevention of Cruelty to Animals, New York, New York, United States of America; 2 Department of Animal Sciences, The University of California- Davis, Davis, California, United States of America; University of Leipzig Faculty of Life Sciences: Universitat Leipzig Fakultat fur Lebenswissenschaften, GERMANY

## Abstract

We extended well-established animal models of human violence paradigms to domestic dogs (*Canis familiaris*) seized from organized dogfighting operations (n = 137). Using standard metrics of frequency, latency, duration, and severity of physical attack and social behavior, we found a pattern of severe intraspecific aggression and alterations in species typical social behavior comparable to that described in the models of violence literature. Behavior was coded from archival video footage of a model conspecific screening test by a technician blind to the categorical behavior severity rating assigned to the dogs on intake. Biting attacks were initiated with short latencies and a dramatically higher prevalence in dogs rated as severe for dog-directed aggression. Furthermore, high intensity attacks involving crushing and shearing bites and guttural growl vocalizations (a heretofore unreported vocalization) were exclusively exhibited by those subjects, and their attacks were directed to vulnerable body regions of the model, including the throat. Social investigation was absent or abbreviated; many individuals in this category failed to investigate the anogenital region of the model conspecific and spent a significantly smaller proportion of the test sniffing the model. Conversely, the comparator group from the same lines of domestic dogs showed normal social behavior toward the model conspecific despite their directional selection and life history. Our findings are the first to quantify the topography of extreme intraspecific aggression in domestic dogs, broadening our understanding of the social behavior of *C. familiaris*. In addition to contributing to basic science, our findings also support the use of expert ratings to categorize extreme intraspecific aggression in fight-bred lines. That finding is of applied value to shelter professionals making outcome decisions, and to legal professionals who require objective evidence grounded in accepted scientific paradigms when considering the prosecution of organized dogfighting cases.

## Introduction

*“…all species manage as a rule to settle their disputes without killing one another; in fact, even bloodshed is rare.” -Tinbergen, 1968 pp1412*

Tinbergen’s assertion notwithstanding, extreme intraspecific aggression, up to and including fatal conflict, is well documented in a range of mammalian species, including wild canids [1,2]. In wolves, Schenkel described 3 categories of intraspecific fighting: strict intolerance, special privileges, & minor within-group conflicts. Strict intolerance is the most injurious type of fighting, characterized by an insensitivity to signals or cues from the losing opponent, with the attack ending only when the loser flees or dies [3]. Work with dingoes suggests qualitatively different forms of intraspecific aggression – one lethal the other generally not – also exists in this canid species: dingoes kill conspecifics by targeting vulnerable body regions with crushing bites that damage internal organs, while less intense fights center on the frontal body regions and involve lacerations & punctures [4]. Domestic dogs kept as pets, too, are known to attack and severely injure or kill conspecifics, though the precipitating factors and patterns of attack are not understood [5].

On the other hand, severe aggression is reportedly very rare in free ranging dog populations [6] and among village dogs [7]. Even during artificially staged contests for resources, domestic dogs were reported as ‘weakly aggressive’ [8]. Such accounts are in keeping with an extrapolation of the behavioral components of the ‘Domestication Syndrome’ [9] namely, applying the reduction in heterospecific aggression to all aspects of domesticated dogs’ temperaments. This results in the perception of domesticates as generally less aggressive and more infantilized [10] compared to their wild progenitors who, being more natural, are more ‘red in tooth and claw’ [11]. However, recent work has challenged the domestication syndrome generally and as regards dog behavior specifically [12,13].

Such views are also at odds with the accepted understanding of natural and artificial selection processes. Natural selection favors the ritualization of agonistic interactions as a mechanism to reduce physically damaging encounters that are costly to fitness [14]. That was Tinbergen’s thesis in the paper quoted above: ritualized signals allow conspecifics to contest access to or holding of resources while obviating the need for risky physical attacks and ‘bloodshed’ [15]. This viewpoint is frequently employed in applied dog behavior contexts, where ritualization is used as an explanatory means of pathologizing physical aggression, e.g., dogs escalating to physical attack are ‘abnormal’ unless they fear for their own safety [16].

Yet, a hallmark of domestication is the relaxation of natural selection, via a buffering against fitness consequences by human caretakers [17]. Just as the process of domestication relaxes natural selection against morphologically deleterious traits, e.g., white coloration patterns that are less cryptic [18], it also relaxes natural selection acting on behavior traits [19]. Comparative studies of domestic dogs, wolves, jackals, and wolf dog hybrids provide evidence that supports the relaxation of ritualized signaling and an increase in the incidence of physical aggression in domestic dogs. Domestic dogs have fewer facial expressions compared to wolves [20] thus rendering them less able to signal agonistic intent visually – although a larger vocal repertoire may fill that signaling gap [21]. Across the first year of life, domestic dogs engage in aggressive interactions significantly more frequently than wolves – on a par with golden jackals, a canid species with a simpler social structure than wolves [22]. Escalations to aggression occurred in response to a variety of precursor behaviors from the other actor, including prosocial signals used to solicit play [22]. Recent work on dominance relationships in domestic dogs reported steeper hierarchy distances and a higher frequency of aggression in dog dyads compared to wolf dyads [8]. Furthermore, escalation to physical aggression is a common problem for pet owners in multi-dog households. A recent representative-sample, survey-based study found that inter-dog aggression was the most commonly reported issue related to aggression in owned pet dogs [23]. Together, the literature suggests a relaxation of the natural selection pressure that favored ritualization, resulting in more variability in aggressive tendencies within domestic dogs compared to their wild progenitor canid species [24], at least in the context of owned dogs under human care [6].

Another hallmark of domestication is the imposition of artificial selection, commonly in the form of directional selection for a trait of interest [25]. With some notable exceptions, e.g., non-working purebred Rottweilers [26], laboratory-bred lines of anxious pointers [27], and working lines (described below), there is little systematic artificial selection on behavioral phenotypes in domestic dogs. Instead, artificial selection tends to focus on physical traits deemed desirable, summed up as “capricious” selection with “no real functional purpose” and little to no selection on behavioral traits [28]. In this process, behavioral traits and their relationships can be uncoupled by artificial selection, further eroding any ‘syndrome’ produced during initial domestication [29]. Many dogs don’t experience any selective pressure because they are removed from the gene pool regardless of their behavioral or morphological traits [30]. This means that, generally, there is little or no artificial selection acting to compensate for relaxed natural selection on traits such as the ritualization of aggression. This allows for a higher frequency of non-ritualized aggression in the population.

Directional selection can act on this variability to increase the frequency of trait aggression. This process occurred in the later phase of the farmed fox program, where a high-aggression line was created by applying directional selection *for* defensive aggression to humans [31]. And, unlike the pet dogs described above, working lines commonly undergo intense selection for morphological and behavioral traits related to task performance [32]. Directional artificial selection can produce animals with hypertrophied, maladaptive traits but who are maintained in the population via relaxed natural selection [33]. Lines of domestic dogs bred for organized dogfighting (colloquially referred to as ‘fight-bred’ lines) undergo such directional selection for intraspecific aggression to a degree that results in significant physical injuries and interferes with mating. Fight-bred dogs are also repeatedly exposed to training and/or official fighting matches that promote aggression via experiential learning [34]. This creates a situation where intraspecific aggression is more frequent in the fight-bred population compared to non-selected lines.

However, despite directional selection and experiential learning, many individuals are *not* aggressive towards conspecifics. This phenotypic variability is consistent with the complexity of inheritance and expression of behavioral traits, which are generally polygenic involving many genes of small effect [35,36], including aggression [37]. This is summed up colloquially by a dogfighter and breeder who said to an undercover investigator: “You’re lucky if you get one game dog in a litter” (Mills, pers. comm.). Conversely, a subset of individuals in these selected lines *do* exhibit extreme intraspecific aggression.

It is critical, therefore, to be able to screen directionally selected lines for intraspecific aggression in a way that maximizes lifesaving and placement of non-aggressive individuals, while reliably detecting those who are unsafe to place in the community. Recently, we reported that life-size plush model dogs are effective screening tools for extreme intraspecific aggression in fight-bred lines. Within-individual behavior to the model was consistent with behavior to a live conspecific as categorized by the ratings of expert behavior professionals [38]. As discussed in our previous paper, the findings were at odds with some of the shelter behavior research assessing the validity of model dogs; this raised the question of why our findings were so consistent compared to other studies [38]. Because our previous study used only subjective ratings to categorize behavior to the model and live conspecifics, unconscious rater bias could not be ruled out as a possible explanation for our findings.

Therefore, we sought quantitative approaches to the analysis of aggression in animals. Rodent models of human violence also employ lines directionally selected for high trait aggression to conspecifics [39]. The higher frequency of trait aggression facilitates the development of pathological aggression thought to be an analog to human violence, thereby enabling research assessing the causes of the extreme aggression and the efficacy of interventions to modify the behavior [37]. Such aggression is termed ‘impulsive’, and the development of impulsive aggression requires repeated exposure to fighting opportunities [40].

Employing resident-intruder tests, which mimic a biologically salient encounter likely to elicit aggression, models of violence paradigms quantify the intensity and severity of intraspecific aggression using standardized metrics [41]. Impulsive aggression, described as a ‘first act, then think’ approach ( [42] p. 497), is characterized by short attack latencies, a concomitant reduction in processing of social signals, and an increased severity of physical attack compared to physical attacks from ‘normal’ conspecifics of the same directionally selected line [42]. Attack severity is measured by the frequency of physical attacks and the location of attack on the victim’s body. Attacks differ not only in the eliciting stimulus and frequency (who is attacked and when), but also in how long the attack persists and what parts of the body are targeted, with vulnerable body regions such as the neck and flank preferentially targeted [37].

We hypothesized that model dogs were effective screening tools for extreme intraspecific aggression in the fight-bred population [38] because of a higher incidence of impulsive aggression in these lines. We predicted that, similar to animal models of human violence, impulsively aggressive individuals would exhibit short latencies to attack, would unleash intense attacks, and would demonstrate an insensitivity to the social cues of their (model) victims. We further predicted that these objective measures would relate to expert ratings used to categorize extreme intraspecific aggression in fight-bred lines.

The study reported here used archival video from a large sample seized from organized dogfighting operations to test our hypotheses and broaden our understanding of the social behavior of *C. familiaris.* Our study is the first to quantify the topography of extreme intraspecific aggression in domestic dogs. Our findings supported our predictions and extend well-established paradigms from animal models of violence; they also support the use of expert ratings and have applied value for shelter and legal professionals.

## Materials and methods

### Ethical note

This study was a retrospective records-based analysis of videos recorded during normal intake behavior evaluation procedures. The housing & husbandry, enrichment, behavioral & medical care, and assessment of animals complied with ASPCA internal protocols and procedures in place for national animal cruelty cases at the time of intake; they met or exceeded all applicable local, state, and/or federal statutes. Model dogs are used as first-pass screening tools when assessing aggression to avoid the ethical concerns raised by exposing living stimulus dogs (colloquially called “helper dogs”) to potentially violently aggressive conspecifics (for details see [38]).

### Subjects and evaluations

Behavior was coded from archived video footage of the model dog subtest (hereafter, “subtest”) section of forensic behavior evaluations carried out on dogs seized from 8 organized dogfighting cruelty cases. Cases were selected randomly. Behavior evaluation methods are described in our previous publication [38]. Briefly, behavior specialists conducted the evaluations, and each dog was assigned a categorical rating denoting the severity of behavior concern(s), which was abbreviated by a letter ‘grade’ ranging from A to D (the “Grade Category”). The categories were as follows- A: No behavior concerns; B: Mild behavior concerns; C: Moderate behavior concerns; D: Severe behavior concerns.

Behavior concern ratings can be due to Fear, Aggression to Dogs, Aggression to People, High Arousal, or ‘Other’ and may be simple (only one concern reason is selected) or complex (multiple concern reasons are selected). Inclusion criteria for dogs assigned ‘D’ grades were age (as described below) and intraspecific aggression listed as a reason for the behavior grade – either alone or in conjunction with other reasons. In the authors’ experience, intense human-direct aggression is rare in directionally selected lines of dogs from organized dogfighting cases and was absent in the study sample reported here (note this observation is in line with the independence of con- and hetero-specific aggression in laboratory strains differentially selected for aggression [43]).

A total of 92 video clips (80% of eligible dogs) were quasi-randomly selected to ensure at least 70% coverage of D Grade Category dogs per case (female and male, n = 46 each). A random sample of A, B, and C Grade Category test dog videos (n = 15 per category for a total comparator n = 45; female n = 21 male n = 24) were also included as a comparator group to confirm that extreme aggressive behaviors were confined to dogs assigned to the severe Grade Category. There was no behavior concern inclusion requirement for the comparator dogs as, by definition, some had no or only mild concerns and, in the authors’ experience, intraspecific aggression is generally pronounced or absent in adult dogs in these populations, i.e., it is not normally distributed, especially in adults.

Only adult dogs (1–7 years of age) were considered for this study. Puppies are never tested with the model dog, so their behavior evaluations were excluded from this analysis. Because fighting experience is thought to increase the prevalence of extreme aggression in fighting dogs [44], we excluded juvenile dogs (n = 8). Senior dogs (n = 4) were also excluded as their behavior could be limited by mobility issues or exacerbated by fighting experience. Including only adult dogs reduced confounds from age/experience effects.

### Video coding of behaviors

Dogs were tested for intraspecific aggression with a commercially produced adult-size, plush yellow lab model dog (Melissa and Doug™), that was constructed without genitals or any obvious secondary sex characteristics. As described in our previous paper, the test dog was turned away from the entrance to the test arena prior to the primary handler’s entrance with the model dog. They were gently held in place by the assistant handler placing their legs on either side of the test dog’s waist just in front of the dog’s hips and grasping the collar on either side of the test dog’s head. Once the primary handler entered and was ready, the assistant handler released the test dog by removing their hands and rotating their leg out of the way. Once released, the test dog was able to move freely and interact with the model dog (while on a loose leash, see [38]).

**Fig 1:**
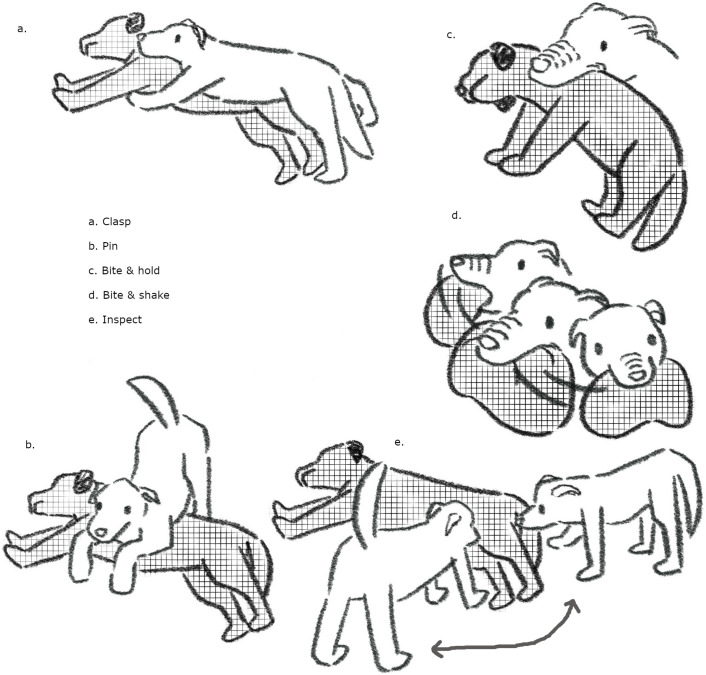
Illustration of select behaviors. This sketch illustrates a sample of behaviors included in the video coding behavior inventory. The model dog is indicated by hatched marks and passive pose. Behaviors shown are a) Clasp b) Pin c) Bite & Hold d) Bite & Shake e) Inspect. See Table 1 for a full list of behaviors and their definitions. Illustrations by Q. Lu.

The video was coded by a temporary research technician (QL) blind to the dogs’ behavior evaluation grades. The technician was not an ASPCA staff member and was not familiar with dogfighting cases nor with the behavior evaluation described in Reid and colleagues [38]. Behavior was coded using the inventory of behaviors and operational definitions presented in Table 1 and illustrated in Fig 1. Whenever possible, behaviors and definitions published in the canid social behavior literature were used to increase comparability; the behavior inventory was compiled following a literature review that identified ethograms used to describe aggressive & conflict behavior in free living and captive wild and domestic canids. A short list of behaviors was compiled by trial coding a selection of videos. Where necessary, modifications were made to definitions to make them appropriate for use with a model conspecific. Some items were created and defined by the authors. Table 1 credits the original paper where the term was defined, unless it was created by the authors for this study. Behavior states were coded as mutually exclusive but could co-occur with point counts of events.

**Table 1:** Behavior inventory used to code the model dog subtest videos.

Behavior	Description	Source	Species
**Bite and Hold**	A gripping bite, where the test dog puts the model dog in their mouth & bites down without releasing, except may ‘re-grip’ where they open and immediately close their jaws.	Authors	Dog
**Biting, fixated**	Test dog is lifted off their front and/or hind feet by the handler, but they continue to grip the model dog with their teeth for at least 3 s. May be clasping the model at the same time.	Authors	Dog
**Clasp**	Actor presses forelimbs around recipient’s torso, ‘holding’ on with their forepaws.	[6]	Dog
**Clasping, fixated**	Test dog is lifted off their front and/or hind feet by the handler, but they continue to grasp the model dog with their forelegs for at least 3 s. No biting of model.	Authors	Dog
**Clasp and thrust**	Actor places his forepaws around recipient’s torso and thrusts his/her pelvis.	[6]	Dog
**Freeze**	General rigidity of the body, with exception of the tail, and no staring towards the recipient.	[45]	Dog
**Head Over**	Actor approaches recipient and places his/her head over recipient’s back or neck.	[6]	Dog
**Inspect**	Lick and/or sniff anogenital area of a conspecific.	[46]	Wolf
**Pinning**	Actor places chest over recipient body with weight shifted to two front paws, so as to prevent recipient from raising up, may pin with full body weight by lying on recipient.	[6]	Dog
**Ride Up**	Mount or jump on a conspecific from behind or the side, in an aggressive posture (tense body, raised tail, may growl, piloerection). May have one or two forepaws on the recipient, or clasp.	[46]	Wolf
**Searching**	Test dog repeatedly pokes or pushes at model dog’s body with open or closed muzzle, while model is on the ground, without biting. May walk on, paw at, or ‘pin’ model repeatedly.	Authors	Dog
**Sniff**	Sniff part of a conspecific’s body, excluding anogenital area.	[46]	Wolf
**Stand Over**	Actor stands over recipient body, with 4 feet on the ground, a conspecific that is lying down so as to prevent recipient from raising up.	[46]	Wolf, Dog
**Stare**	Intense ﬁxating look towards recipient with tense body, for a minimal duration of 2 s.	[45]	Dog
**T Position Dominant**	Stand as dominant in the T-formation, as the horizontal cross of the T, facing the chest of a conspecific.	[46] (includes an illustration)	Wolf
**Tail Flagging**	Tail held in High position (see Postures) tip is slowly moved only a few degrees off midline from side to side. When not moving, tail may appear to vibrate.	Authors (but also ‘tail rattle’ [37])	Dog, Mouse
**Point Counts**	**Description**	**Source**	**Species**
**Bite/Nip**	Taking any part of the recipient’s body between the jaws with sufﬁcient pressure that could cause damage to the recipient. Record count as 0 if not used. A bout of biting/nipping includes all bites/nips w/in 5 s of the last bite/nip.	[45]	Dog
**Bite and shake**	The test dog vigorously shakes the model dog by moving their head side to side rapidly, while gripping with their teeth – either during a Bite and Hold or Fixated Biting event. Each pause begins a new count.	Authors	Dog
**Break Stick**	Handlers used a break stick to remove the actor from the recipient.	Authors	Dog
**Growl**	Low-pitched rumbling, fairly monosyllabic vocalization from the dog’s throat	[45]	Dog
**Guttural Growl**	As with Growl, but harsher and with greater amplitude. May pause for up to 3 s within same bout of growling.	Authors	Dog
**Lunge At**	Actor moves abruptly and rapidly towards recipient, while staring at him/her but without making physical contact, may be combined with air snap.	[6]	Dog
**Knockdown**	Hit or push down a conspecific by pushing with body or with paws.	[46]	Wolf
**Regrip**	The test dog opens their jaws and re-bites the model dog, without changing their body position & within 5 s of releasing, during a Bite and Hold or Fixated Biting event.	Authors	Dog

For some analyses, related behaviors were assigned to categories (hereafter “Combined Behavior Group” abbreviated CGB) as follows: Avoid (single behavior); Biting behavior group = Bite, Bite & Hold, Biting Fixated, Shake Bite, Regrip; Clasping behavior group = Clasping, Clasp & Thrust, & Clasp, Fixated; Knock Down (single behavior); Pinning (single behavior); Posturing behavior group = Head Over, Ride Up, Stand Over, Stare, T-Position Dominant; Prosocial behavior group = Inspect, Sniff; Vocalizing behavior group = Growl; Guttural Growl).

### Metrics and statistical tests

Unless otherwise stated, analyses were conducted using base R version 4.3.3. Specific packages are listed with the test they were used to run. Test duration was compared across severity categories using a Kruskal Wallis test.

### Prevalence of behaviors across grade categories

The prevalence of behaviors in dogs belonging to different Grade Categories was calculated as follows: for every CBG, each subject was counted a single time (assigned a 1) if they exhibited any behavior in that combined group during the subtest, regardless of the number of behaviors or the frequency/duration of bouts. Dogs exhibiting multiple behaviors from a given behavior group were counted only once in the combined data. For example, a dog that exhibited both Bite and Bite & Shake was counted once in the Biting behavior group. Pearson’s chi-square tests were used to test for differences in the prevalence within Grade Category for each CBG, except where the expected count was < 5; in those cases, a Fisher’s Exact Test was used. Where significant differences existed, post-hoc testing was done using the chisq.posthoc.test package in R, with a Bonferroni correction for multiple comparisons.

### Attack intensity: Frequency & duration, latency, location

Attack intensity was determined by the frequency & duration of attacks, latency to attack, and attack locations. Sensitivity to social cues – another indication of pathological aggression [47] – was determined by the prevalence & duration of social investigation and the latency to Inspect the anogenital region of the model.

The time at which the handler released the test dog was recorded (T_0_) and this T_0_ demarked the beginning of the model dog subtest. Subtest duration was calculated as time at model dog removal from test arena (T_end_) minus the time at the start of the interaction test (T_end_ - T_0_).

To examine the frequency of occurrence among individuals that exhibited a given behavior, the number of bouts was summed for all animals within a given Grade Category, across all behaviors within a given combined behavior group (e.g., Total Bites includes Bite and Release, Bite and Hold, Bite and Shake, etc.). The total number of bouts was then divided by the prevalence (in subjects in that Grade Category, as described above), to provide an average total count of bouts weighted for prevalence for each combined behavior group among individuals of a given Grade Category.

For each behavior coded as a duration, start time was subtracted from stop time to determine the number of seconds (s) the behavior was exhibited during a bout. Bout durations were summed to determine the Total Duration (s) for each dog-behavior combination. Because the model dog test duration varied between subjects, Total Duration was converted to a proportion of test time as follows: Proportion of Test Time = Total Duration/ (T_end_ – T_0_) for each dog-behavior combination. Because these were continuous proportions, beta regressions were used [48,49] to determine the relationship of Grade Category to the Proportion of Test Duration for particular behaviors of interest, specifically physical attack and social behavior. Beta regression models were run using the betareg package [50] in r, with dispersion parameter (*ϕ,* fixed or variable) and overall model fit determined using the boot, lmtest, and emmeans packages as described in Douma & Weedon (2019). Tukey familywise error corrections were applied to post-hoc pairwise contrasts.

The latencies to knockdown, bite, and inspect the model conspecific were calculated as the number of seconds elapsed from T_0_ until the occurrence of a given behavior. A time of 180 s was assigned to test dogs that did not knockdown, bite, or inspect the model, as that duration exceeded the maximum latency across all subjects (Table 2). Kruskal Wallis rank sum tests were used to test for differences in latencies to attack and inspect the model across subjects in different Grade Categories. Where significant differences existed, post-hoc testing was done using the dunnTest() function with a Bonferroni correction for multiple comparisons in the FSA package v 0.9.5 [51].

**Table 2:** Calculations used for latencies to bite, knockdown, and/or inspect the model dog.

Event	Description and Calculation	Formula
**Start of Clip**	Always 0:00:00 with variable duration until T_0_	
**Test Dog Released**	Handler pivots allowing the test dog to move out of the corner.	T_0_
**Model Dog Removed**	The model dog is removed from the test area by the handler.	T_end_
**Latency to Bite**	Time elapsed (s) from test dog release to first bite to model. If no bite occurred, latency = 180 s	T_bite-_T_0_
**Latency to Inspect**	Time elapsed (s) from test dog release to first sniff of model’s anogenital region. If no inspect occurred, latency = 180 s	T_inspect-_T_0_
**Latency to Knockdown**	Time elapsed (s) from test dog release to first knockdown. If no bite knockdown occurred, latency = 180 s	T_knock-_T_0_

**Fig 2:**
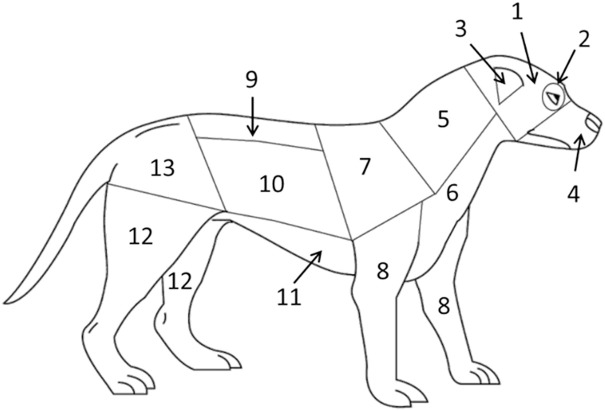
Canine body region map used to record location of bites to the model dog. The 13 regions are: 1 Dorsal and lateral head; 2 Eye and periorbital region; 3 Pinnae; 4 Muzzle and oral mucosa; 5 Dorsal and lateral neck; 6 Ventral neck and chest; 7 Scapular region; 8 Front legs; 9 Thoracic and lumbar spine; 10 Lateral thorax and abdomen; 11 Ventral thorax and abdomen; 12 Hind legs; 13 Pelvis and tail. For this study, we combined the head and orbital (1 & 2) and the spine, thorax, and abdomen (9, 10 & 11) regions when coding bites to the model dog from video. Reproduced from Miller and colleagues [44] under the terms and conditions of the Creative Commons Attribution (CC-BY) license.

Video clips that contained physical attacks (the test dog delivered bites to the model dog) were flagged at the end of the initial behavior coding. Those clips were then re-watched and the body region where each bite was delivered to the model dog was recorded. Bite regions were defined using the region map from Miller and colleagues [44] shown in Fig 2. We combined regions 1 & 2, and also combined regions 9, 10, & 11, into two larger regions based on ability to distinguish regions reliably from video, as described in Fig 2.

Descriptive statistics were calculated for the regional distributions of bites to the model (number of bites~body region), to facilitate comparison to the published distributions of patterns of injury and scarring in dogs [52] and of wound patterns reported in studies of conspecific conflict & killing in dingoes [4].

## Results

Unless otherwise noted, the sample size for all results is n = 92 D severity grade dogs and n = 45 comparator dogs (15 each of A, B, and C severity grades). All post hoc tests report the corrected p-values after adjusting for multiple comparisons.

### Subtest duration

The subtest duration was similar across severity grade categories. Subtests were somewhat shorter for dogs with no behavior concerns (A grade, median 55 s) and somewhat longer for dogs with intermediate behavior concerns (C grade, median 75 s) but there was no significant difference in test duration (Kruskal-Wallis x2 = 3.01, df = 3, p = 0.39) across severity categories (see Table 3).

**Table 3:** Model dog subtest durations (s) across severity categories.

Grade:	Median	Min	Max	IQR
**A**	55	39	107	26
**B**	63	50	112	22
**C**	75	44	134	25.5
**D**	66	12	175	51.25

### Prevalence of behaviors

All prevalence figures are rounded to the nearest percentage. Individual behaviors are reported in S1 Table Combined behavior groups are reported in Table 4.

**Table 4:** The prevalence of behavior groups across behavior concern severity categories. The A:C line reports the total number and the average percent of dogs in the comparator group.

	Grade Category	Number of Dogs Exhibiting Behavior	Percent of Dogs Exhibiting Behavior
**PROSOCIAL** ^a^	A	15	100
	B	15	100
	C	12	80
	*A:C*	*42*	*93*
	D	72	78
**AVOID** ^b^	A	6	40
	B	5	33
	C	0	0
	*A:C*	*12*	*27*
	D	0	0
**POSTURING** ^**c**^	A	9	60
	B	7	47
	C	14	93
	*A:C*	*30*	*67*
	D	72	88
**KNOCK DOWN** ^d^	A	4	27
	B	3	20
	C	12	80
	*A:C*	*19*	*42*
	D	81	88
**PINNING** ^e^	A	3	20
	B	1	7
	C	3	20
	*A:C*	*7*	*16*
	D	54	59
**CLASPING** ^f^	A	2	13
	B	3	20
	C	11	73
	*A:C*	*16*	*36*
	D	48	52
**VOCALIZING** ^g^	A	0	0
	B	0	0
	C	1	7
	*A:C*	*1*	*2*
	D	28	30
**BITING** ^h^	A	1	7
	B	0	0
	C	4	27
	*A:C*	*5*	*11*
	D	74	80

The Number column shows the count of dogs within that category exhibiting behavior from each group. The Percent column shows the number of dogs as a percentage of the total number of dogs in that severity category. Details for individual behaviors are reported in S1 Table.

a Sniff; Inspect.

b Avoid.

c Head Over; Ride Up; Stand Over; Stare; T Position Dominant; Tail Flagging.

d Knock Down.

e Pinning.

f Clasping; Clasp and Thrust; Clasping, Fixated.

g Growl; Guttural Growl.

h Bite; Bite and Hold; Shake Bite; Biting, Fixated; Regrip.

### Social investigation

Social investigation differed significantly across grade categories (Fisher’s Exact Test p = 0.036). All A and B grade dogs sniffed the model (in non-anogenital areas), whereas just over one-quarter of C (27%) and D (26%) grade dogs failed to sniff the model during the subtest. Almost two-thirds (61%) of D grade dogs failed to inspect the model dog’s anogenital area, compared to a quarter of C grade dogs (27%). Conversely, the majority of A and B grade dogs (80% and 93%, respectively) investigated the model dog’s anogenital area during the subtest. Combining these two behaviors, all A and B grade individuals sniffed, investigated, or both (100%) compared to just over three-quarters of C and D grade individuals (80% and 78%, respectively) where a fifth of individuals exhibited no social exploration toward the model dog.

### Avoid

Avoiding the model dog differed significantly across Grade categories (Fisher’s Exact Test p < 0.0001). No D or C grade dogs avoided the model, whereas 40% of A grade dogs and 33% of B grade dogs did avoid the model at some point during the subtest.

### Posturing

Posturing differed significantly across Grade categories (Fisher’s Exact Test p = 0.009). Stand Overs were most common in C grade dogs, with one-quarter of subjects standing over the model dog (27%); they were less common in D grade dogs (14%), absent in B grade dogs, and exhibited by two A grade dogs (13%). Head Overs were most common in B and C grade dogs (both 13%), absent in A grade dogs, and exhibited by few D grade dogs (5%). About three-quarters of D and C grade dogs rode up on the model dog (73% and 80%, respectively) compared to about half of A grade (47%) and one-third of B grade dogs (33%). Test dogs rarely stared at the model, but this was most common in A grade dogs (13% A grade, 4% D grade, absent in B and C grade dogs). Only 5 D grade dogs (5%) produced a T Position Dominant stance against the model. Tail Flagging was also rare, confined to a single dog each in the C and D grade categories. Overall, more than twice as many D grade dogs postured during the subtest than did comparator group dogs (88% vs 42%).

### Knockdowns

Knockdowns differed significantly across Grade categories (Fisher’s Exact Test p < 0.0001). The model dog was knocked down by just over one-quarter of A (27%) and ca. one-fifth of B (20%) grade dogs; more than three-quarters of C and D grade dogs knocked the model dog down during the subtest (80% and 88%, respectively).

### Pins

Pins differed significantly across Grade categories (Χ^2^ = 22.4, p < 0.0001). D grade dogs were the only group significantly more likely to pin the model dog than not (Observed vs Expected = 53 vs 40, p = 0.00003). Over half of D grade dogs pinned the model dog (59%), compared to a fifth of C and A grade dogs (20%). B grade dogs pinned the model less than expected (Observed vs Expected = 1 vs 7, p = 0.017) and less than 10% of B grade dogs (7%).

### Vocalizations

Vocalizations differed significantly across Grade categories (Fisher’s Exact Test p = 0.0008), driven by the D Grade subjects. Growling was rare across subjects; 11% of D grade and 7% of C grade dogs growled, and growling was absent in A and B grade dogs. Guttural growls occurred in almost one-quarter of D grade dogs (23%) while absent in C, B, and A grade individuals. Guttural growls are subjectively perceived as low frequency, high decibel vocalizations that are, in our experience, unique to extreme intraspecific aggression. Combining both types of growls, almost one-third of D grade individuals either growled, guttural growled, or both (30%), compared to a single C grade individual (7%) and no B or A grade dogs.

### Clasping & sexual behavior

Clasping and sexual behavior differed significantly across Grade categories (Χ^2^ = 16.39, p = 0.001). Clasping (wrapping and holding the model with front limbs) was exhibited by ca. one-third of D and C grade dogs (34% and 33%, respectively), one B grade dog and two A grade dogs (7% and 13%, respectively). Clasping and thrusting (i.e., sexual behavior) showed a somewhat different pattern – it was most common in C grade dogs, exhibited by just over half the individuals in this grade category (53%), followed by B grade dogs (20%); it was least common in A and D grade dogs (13% and 14%, respectively). Fixated clasping, defined as keeping a clasping hold on the model while being lifted off the ground by the handler, was exhibited by just under one-quarter of D grade dogs (23%), a single C grade individual (7%), and was absent in B and A grade dogs. Combining all forms of clasping, two-thirds of C grade (73%), one half of D grade (52%), one-fifth of B grade (20%) and just over a tenth of A grade (13%) individuals exhibited at least one form of clasping toward the model (see Table 4). Only A grade subjects exhibited a prevalence significantly different from expected (Observed vs Expected = 2 vs 7, p = 0.048).

### Physical attacks

The prevalence of physical attacks differed significantly across Grade categories (Χ^2^ = 61.9, p < 0.0001). Combining all forms of biting, over three-quarters of D grade dogs (80%) delivered bites to the model (significantly more than chance; p = 0.0000), compared to a quarter of C grade (27%) no B grade individuals, and a single A grade dog (7%) as shown in Table 4. A and B grade dogs were significantly less likely than chance to bite the model (p = 0.0002 and p < 0.0001, respectively), while C grade dogs had a non-significant trend for a lower-than-expected prevalence (p = 0.08).

### Attack frequency and duration

Across all bite types (bite/nips, gripping bites, etc.) and subjects, a total of 799 bites were counted. Of these, 92% were delivered by dogs with a D grade rating, 7.9% by dogs with a C grade rating, and 0.1% by a single bite from an A grade rating dog. Therefore, attack intensity is reported only for D and C grade dogs.

The C grade dogs that bit the model (n = 4) delivered a total of 63 bites, across all bite types, for an average of 16 bites per subject. The D grade dogs that bit the model (n = 74) delivered a total of 735 bites, across all bite types, for an average of 10 bites per subject (see S1 Table). Six D grade dogs required a break stick to remove them from the model. Bites with release (Bite), gripping bites (Bite and Hold & Biting, fixated), regrips, and kill bites (Bite and Shake) occurred at a prevalence at least double and up to eight times higher in D grade dogs compared to C grade dogs (Bite 75% vs 28%; Bite and Hold 53% vs 7%; Biting, fixated 35% vs 7%; Regrip 28% vs 7%; Bite and Shake 42% vs 7%).

Planned beta regressions for the proportion of test duration spent attacking could not be made, due to the almost absence of such behaviors in Grade Categories other than D grade (see Prevalence results, above, and Table 5). Of those dogs in the D Grade Category that employed a gripping bite on the model, they spent on average 17% of their test duration in a “Bite and Hold” behavior (proportion of test duration range: 0.04 to 0.98, median = 0.274). Those dogs in the D Grade Category who could not be distracted from biting spent, on average, 9% of the model dog test coded as “Biting, fixated” (proportion of test duration range: 0.037 to 0.877, median = 0.214).

**Table 5:** The prevalence and intensity of attack behaviors exhibited by severely aggressive dogs (Grade D, n = 92) and comparator subjects (Grades C, n = 15).

	Concern Severity Grade	Dogs Exhibited (# of Dogs)	Prevalence (% of Dogs)	Total Count (# of Events or Bouts)	Frequency (Total Count/ Dogs Exhibited)	Average Percent of Test Duration (Avg Total Duration^a^/ Avg Test Duration * 100%)
**Bite**	C	4	26.7	63	15.75	–
	D	74	80	735	9.9	–
**Bite & Hold**	C	1	6.7	6	6	3.3%
	D	49	53.3	98	2	16.6%
**Bite & Shake**	C	1	6.7	11	11	–
	D	39	42.4	173	4.44	–
**Biting, Fixated**	C	1	6.7	1	1	0.3%
	D	32	34.8	46	1.44	9%
**Regrip**	C	1	6.7	4	4	–
	D	26	28.3	129	4.96	–
**Break Stick**	C	0	0	0	0	–
	D	6	6.5	8	1.33	–
**Pin**	C	3	20	3	1	4.7%
	D	54	58.7	83	1.54	11.7%
**Clasp**	C	5	33.3	7	1.4	2.3%
	D	31	33.7	42	1.35	5.3%
**Clasp & Thrust**	C	8	53.3	16	2	11.5%
	D	13	14.1	22	1.69	2.9%
**Clasping, Fixated**	C	1	6.7	1	1	0.2%
	D	21	22.8	24	1.14	2.1%
**Growl**	C	1	6.7	1	1	–
	D	10	10.9	17	1.7	–
**Guttural Growl**	C	0	0	0	0	–
	D	21	22.8	38	1.8	–

The Prevalence column shows the tally of dogs exhibiting a given behavior within that grade category. The Percent column shows the tally of individuals expressed as a percentage of the sample. The Total Count column shows the sum of the number of bouts of a given behavior across all dogs in that sample. The Frequency was calculated as Total Count/ Dogs Exhibited to provide the average number of events or bouts for only those individuals expressing the behavior.

^a^Total Duration (sec) is not reported here. It is the sum of all bout durations for all individuals of that grade category. Total Duration and Average Percent of Test Duration are reported only for state behaviors See S1 Table.

### Use of social cues

**Fig 3:**
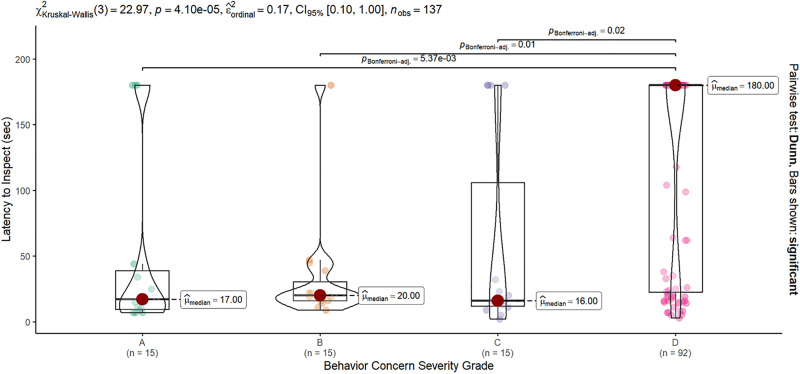
The latency to inspect the model dog’s anogenital region. Latency varied significantly across Grade severity groups (KW X2 = 22.97, p < 0.0001). Dogs rated as exhibiting “Severe” (D grade) behavior concerns had significantly longer latencies to inspect the model dog (median 180 s -– the time assigned when the model was not inspected during the test) compared to dogs from the same fighting cases rated as having “None” “Mild” or “Moderate” behavior concerns (A:C, medians of 17, 20, and 16 s, respectively). Only significant post-hoc contrasts are shown.

The latency to investigate the anogenital region of the model dog differed significantly across Grade Categories (Kruskal-Wallis X^2^ = 22.97, df = 3, p < 0.0001). Dogs rated a “D” grade for severe intraspecific aggression took significantly longer to investigate the model dog compared to all other grade categories based on post-hoc comparisons: A vs D z = −3.32, p = 0.005; B vs D z = −3.11, p = 0.01; C vs D z = −2.95, p = 0.02 all other contrasts non-significant. This difference was driven by the large number of D grade dogs who never investigated the anogenital region of the model (median latency = 180 s, i.e., no occurrence, Fig 3).

**Fig 4:**
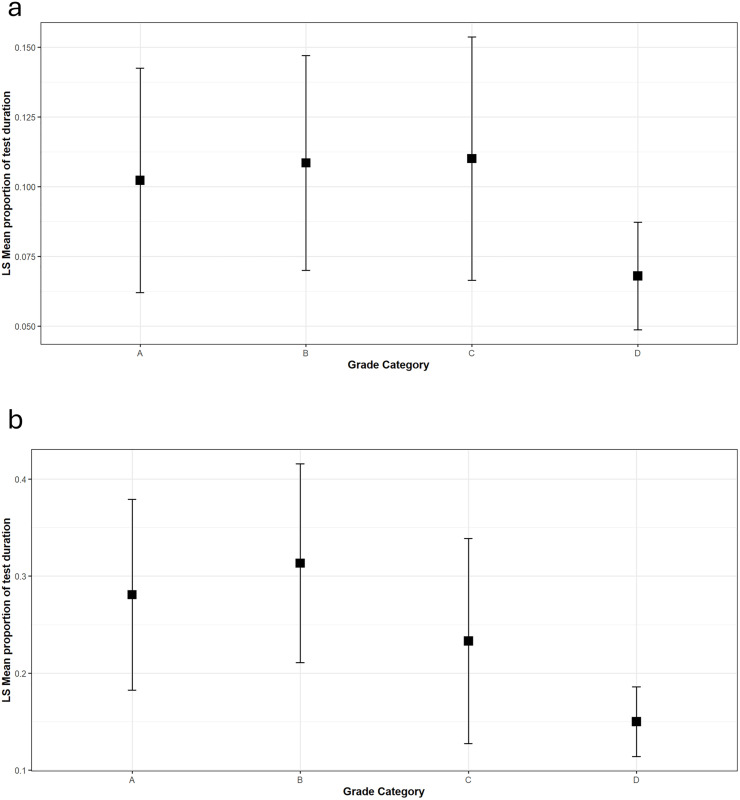
Marginal means and errors for the proportion of test duration coded as a) Inspect and b) Sniff across Grade Categories. Beta regressions found that grade category significantly influenced the proportion of test duration spent inspecting (Χ^2^ = 10.92, df = 3, p = 0.012) or sniffing (Χ^2^ = 23.79, df = 3, p < 0.0001) the model. See text for post-hoc pairwise test results.

The proportion of test duration spent Inspecting the anogenital region of the model dog was related to the behavior concern severity rating (X^2^ = 10.92, df = 3, p = 0.012, Fig 4a). Severely aggressive dogs (D Grade Category) spent a significantly smaller proportion of the test duration Inspecting the model compared to dogs with mild behavior concerns (z ratio = 2.61, p = 0.04); there was a non-significant trend for a reduced proportion of test duration compared to dogs with moderate behavior concerns (z ratio = 2.49, p = 0.061). All other pairwise contrasts were non-significant. The proportion of test duration spent Sniffing (non-anogenital areas) the model dog was also related to the severity of behavior concern in the test dog (X^2^ = 23.79, df = 3, p < 0.0001, Fig 4b). Severely aggressive dogs (D Grade Category) spent a significantly smaller proportion of the test duration Sniffing the model compared to dogs with no (−0.79, z ratio = 3.579, p = 0.002) or mild (−0.948, z ratio = 4.339, p values = 0.0001) behavior concerns. All other pairwise contrasts were non-significant; dogs with moderate concerns (C Grade Category) were intermediate to, but not significantly different from, A, B, or D Grade Cateogry dogs.

### Attack intensity

#### Latency to attack.

**Fig 5:**
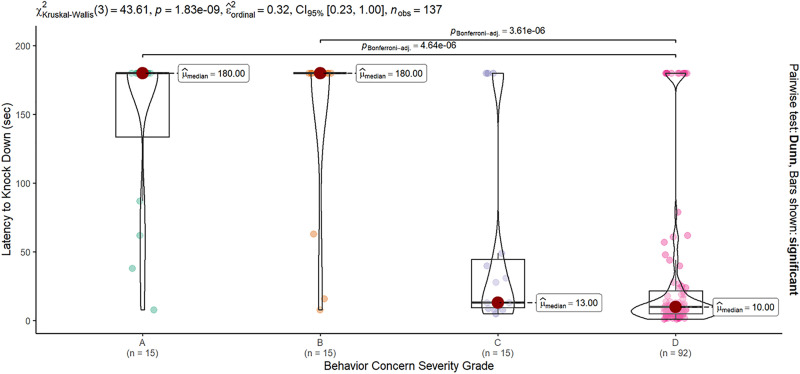
The latency to knock down the model dog. Latency varied significantly across severity groups (KW Χ^2^ = 43.61, p < 0.0001). Dogs rated as exhibiting “Moderate” or “Severe” (C and D Grade Category) behavior concerns had significantly shorter latencies to knock down the model dog (median 10 and 13 s, respectively) compared to dogs from the same fighting cases rated as having “None” or “Mild” concerns (A and B, both median of 180 s – the time assigned when the model was not knocked down during the test). Only significant post-hoc contrasts are shown.

Dogs in all grade categories knocked down the model; the latency to knock-down varied significantly across grade categories (Kruskal-Wallis Χ^2^ = 43.6, df = 3, p < 0.0001). A latency of 180 s was assigned in the absence of a model dog knockdown. A, and B grade dogs had median latencies of 180 s. Three-quarters or more of dogs in those categories did not knock down the model (73% and 80% of A and B grade dogs, respectively). Conversely, over three-quarters of C and D grade dogs knocked the model down (80% and 88%, respectively), with a median latency of 13 and 10 s, respectively (Fig 5). Dogs graded D for intraspecific aggression knocked the model dog over significantly faster than dogs in the A (z = 4.9, p < 0.0001) or B (z = 5, p < 0.0001) Grade Category. There was no significant difference between dogs graded C and any other Grade Category.

**Fig 6:**
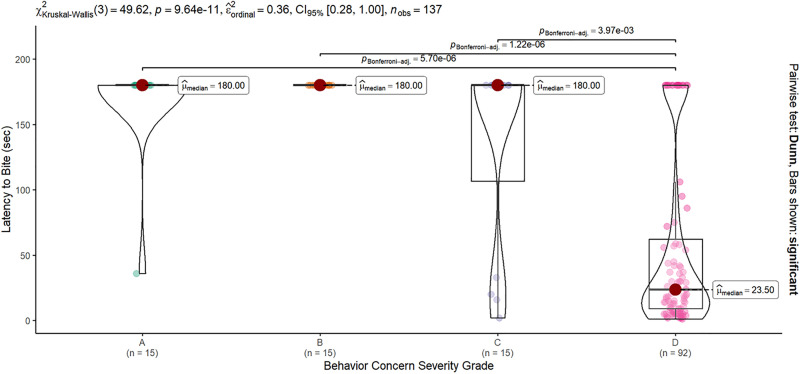
The latency to bite the model dog. Latency varied significantly across severity groups (KW Χ^2^ = 49.62, p < 0.0001). Dogs rated as exhibiting “Severe” (D Grade Category) behavior concerns had significantly shorter latencies to bite the model dog (median 23.5 s) compared to dogs from the same fighting cases rated as having “None” “Mild” or “Moderate” behavior concerns (A:C, all median of 180 s – the time assigned when the model was not bitten during the test). Only significant post-hoc contrasts are shown.

Severity categories also differed significantly in the latency to bite the model conspecific (Kruskal-Wallis Χ^2^ = 49.6, df = 3, p < 0.0001). Dogs rated a “D” grade for severe intraspecific aggression took significantly less time to attack the model dog compared to all other Grade Categories based on post-hoc comparisons: A vs D z = 4.9, p < 0001; B vs D z = 5.2, p < 0.0001; C vs D z = 3.4, p = 0.004 all other contrasts non-significant). A, B, and C grade dogs had median latencies of 180 s. No B grade dogs bit the model conspecific. The sole A grade dog that bit the model did so 36 s into the test. Just over one-quarter of the C grade dogs bit the model, with latencies for those 4 individuals ranging from 2 to 33 s. For the over three-quarters of D grade dogs that bit the model, the median latency to bite was 23.5 s, with a minimum latency of 1 s (Fig 6).

#### Target of attack.

**Fig 7:**
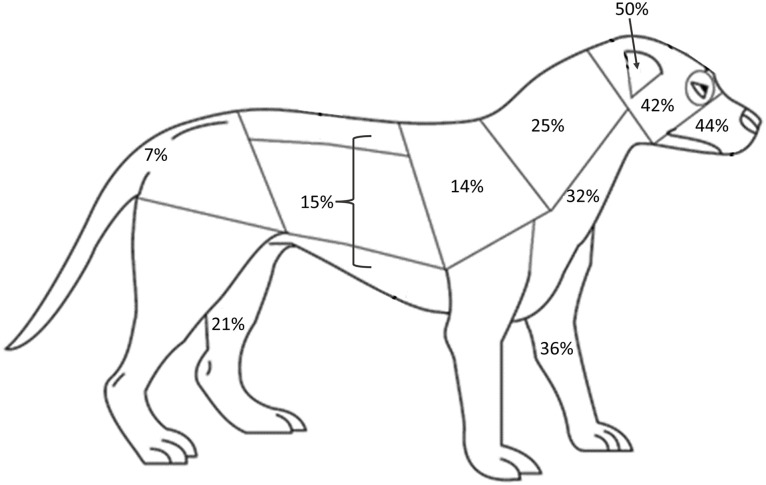
The percent of D Grade Category dogs who bit the model conspecific by body region. Because we were interested in locations targeted by dogs that attacked the model, the denominator used to calculate percentages was 72, the number of D grade individuals (of a total n = 92) that delivered at least one bite to the model during the subtest. Body region image reproduced from Miller and colleagues (2016) under the terms and conditions of the Creative Commons Attribution (CC-BY) license.

Target of attack is reported for D Grade Category dogs only. Bite region data was missing for two D dogs, leaving a sample of n = 90. Of these dogs, 72 individuals delivered at least one bite to the model conspecific. The primary attack location was the model’s face (ears 50%, muzzle 44%, head 42%). The forelegs and throat were the next most prevalent region based on percent of individuals biting (forelegs 36%, throat 32%). A quarter of subjects bit the model’s neck (25%), while a fifth bit the model’s hindlegs (21%). Roughly the same portion of subjects bit the combined back/flank/abdomen as bit the shoulder region (15% and 14%, respectively). Less than 10% of subjects bit the model’s rump/tail region (7%). See Fig 7 for body region schematic.

## Discussion

Our study sought to better describe the topography of aggression using quantitative metrics applied to archival video footage from over 100 dogs seized from organized dogfighting cases. In a previous study we found that a subset of dogs from such differentially selected lines exhibited extreme intraspecific aggression, based on categorical behavior grades assigned by expert raters during a standardized behavior evaluation (as described in Reid et al., 2022). Although our previous work validated behavior to the model dog against behavior to same- and opposite- sex live conspecifics, the ratings are subjective and could, therefore, be subject to implicit rater biases.

Here, we further validated the expert ratings against quantitative metrics closely mirroring those used in thousands of studies on animal models of violence. We showed that dogs rated as severely aggressive (Grade Category = D) exhibited a higher prevalence, frequency, and duration of behaviors related to physical attacks on conspecifics, and they were quicker to mount attacks compared to conspecifics undergoing the same selection pressure but who were not rated as severely aggressive (Grade Categories A:C). Additionally, the subset of dogs exhibiting severe aggression also demonstrated an insensitivity to social cues, as measured by the persistence of their attacks despite visual, tactile, and chemosensory cues indicating the model was not a conspecific, and by the absence of or significant delay in typical canid social greeting behavior. Our findings provide strong evidence that categorical ratings, applied by expert behavior professionals, reflect objective differences in the aggressive behavior of test dogs evaluated.

Our results also indicate that a subset of D grade dogs exhibited a behavioral phenotype that strongly overlaps with “impulsive” or “pathological” aggression described in studies of rodent models of human violence. There, too, lines underwent artificial selection for high trait aggression resulting in populations with a higher frequency of intraspecific aggression compared to wildtypes [39]. Experiential learning was used as a manipulation to elicit ‘pathological’ (aka violent) aggression, and – despite selection and experience – only a subset of the artificially selected line developed pathological aggression [42]. The subset of violently aggressive individuals exhibit patterns of behavior that are qualitatively and quantitatively different compared to normal species-typical aggression, including shortened latencies to attack, heightened severity of attack including altered patterns of attack to preferentially target vulnerable body regions [37], and an insensitivity to social cues from the victims of the attack [47].

For impulsively aggressive dogs, the visual cue of the model seems sufficient to elicit a sustained attack. Species recognition likely employs a multimodal composite signature, meaning dogs can use a single modality of the multiple available to recognize the model as a conspecific. For example, one study reported visual and auditory agonistic signals were redundant in domestic dogs [53], meaning either one is sufficient on their own to convey species-level information. This means that visual cues alone, used from a distance for species recognition, could trigger physical attack in impulsively aggressive individuals [53]. Impulsive aggression could also explain why many of the D Grade Category dogs attacked the model for extended periods – and some needed to be physically removed from the model – because this type of aggression is characterized by a lowered responsiveness to social signals. The rodent paradigms assess sensitivity to social cues by recording attacks on female conspecifics – which are abnormal because territorial males don’t attack females in intruder tests – and on anesthetized conspecifics who are not giving any agonistic cues, posturing, responding to the resident, etc. [54]. Here, attacking the stuffed model dog provides evidence akin to that of the rodent paradigms, though our metrics of social sensitivity were necessarily different. This is because, if the test dogs were responsive to social cues, they would be unlikely to attack or to persist in attacking an inanimate stuffed model dog.

Dogs typically examine the muzzle, inguinal, and anogenital regions of novel conspecifics, gathering additional information, such as sex, reproductive status, etc. from the chemosensory cues [55]. In one study at dog parks, 91% of dogs investigated the anogenital/inguinal area of incoming dogs for a significant portion of the incoming dogs’ first minute in the park, before shifting to other behaviors and increasing solitary behavior [56]. Similarly, dogs generally approach model conspecifics of various degrees of realism and investigate the model before losing interest [57]. Here we found the subset of dogs rated as severe for intraspecific aggression exhibited significant alterations to social behavior compared to the other fight-bred dogs in our sample. Almost two thirds of them never investigated the anogenital region of the model dog, an absence uncommon in the comparator group. Further, the comparator dogs investigated the model’s anogenital region soon after seeing the model, as evidenced by short latencies to inspect – consistent with species typical social behavior and information gathering. Furthermore, a quarter of C and D grade dogs did not sniff the model dog in non-anogenital areas during the subtest, whereas one hundred percent of A and B grade dogs sniffed the model. Extremely aggressive dogs spent a significantly smaller proportion of the test duration sniffing the model compared to dogs with no or mild behavior concerns, with dogs rated as having moderate concerns intermediate. On the other hand, the latency to bite the model was significantly shorter in D grade dogs compared to the comparator dogs (including those rated as having moderate behavior concerns), suggesting they attacked the model without first gathering social information beyond the visual cue. And, they persisted in attacking for a substantial proportion of the subtest, up to and including requiring a break stick to interrupt a gripping bite, suggesting they failed to register or process the model’s tactile and chemosensory cues, which should influence aggressive responses [58]. Taken together, these data are consistent with altered processing of or responsiveness to social information in fight-bred dogs exhibiting extreme intraspecific aggression.

Over three-quarters of dogs assigned a D grade bit the model conspecific. With the exception of a single C grade dog, they were the only test subjects to deliver shake-bites, aka species-typical ‘kill bites’ [7]. Over half of the dogs rated as D for intraspecific aggression delivered crushing bites to the model, which are also hallmarks of intense attacks in canids [55]. Crushing bites coupled with shaking do extensive damage to tissues, organs, bones, etc. and are common in canids killed by conspecifics [4]. During dyadic conflicts, combatants target and defend vulnerable body regions, resulting in characteristic postures and maneuvers of species-typical fighting that often thwart an aggressors ability to land a bite on their preferred target [59]. Because the model in our study could not defend itself from attack, it is possible the attackers’ distribution of bites reflects a true preference for specific body regions – such as the vulnerable throat – which are normally defended during a fight and so less frequently damaged. For example, comparisons between injury patterns from live dingoes attacked by a conspecific and dead dingoes killed by a conspecific showed distinct differences in the body regions sustaining damage [4].

However, it is also possible the bite distribution recorded in our study is, instead, an artifact. The test dog and model dog approach head on, so bites to the head/muzzle/ears could reflect a ‘bite the first available area’ strategy. Similarly, aggressive test dogs frequently knocked over the model, and so bites to the throat could occur just due to the position of the model. It is worth noting, however, that Intarapanich and colleagues (2017) reported that 58% of dogs from suspected organized dogfighting had scars on the throat region, versus just 14% of medium sized dogs involved in a spontaneous fight with a similarly sized conspecific, and scars on the throat are reportedly common in fighting dogs [60]. Similarly, 30% of dingoes killed by conspecifics had injuries to their throats, versus 5% that were injured in a fight but survived [4]. This seems to suggest the ventral neck (throat) is indeed targeted by canids exhibiting extreme intraspecific aggression, including fight-bred dogs. In line with previous research on patterns of scarring where the majority of individuals have scars on their forelegs [44,52], the forelegs were also targeted by the subjects in this study at a rate similar to the throat. This may support anecdotal reports of fighters selecting or training for ‘leg dogs’ that preferentially target the forelegs of their opponent.

Although it is common to claim that fight-bred dogs have altered social communication because they do not signal intent before initiating their aggressive attacks (by way of strategy), we found that almost 90% of D Grade Category dogs did posture during the subtest, a higher prevalence than the comparator group. More likely than selection removing agonistic signaling behaviors from fight-bred dogs’ behavioral repertoires, it may be that short attack latencies result in reduced signaling by virtue of shortening or skipping precursor behaviors that comprise a graded aggressive response [61]. Although this may be of little interest from an applied perspective – because, functionally, it means that it is still challenging to predict when an attack is imminent – it is of importance from the perspective of a basic understanding of the social behavior of domestic dogs (i.e., an inability to signal is different from a lack of signaling incidental to rapid attack). Furthermore, domestication alters thresholds rather than removing behaviors from the species’ repertoire [62] and ethological studies have demonstrated that pitbull-type dogs have *more* facial expressions compared to other breeds such as German shepherds [63]. Our findings serve as a cautionary tale of ‘just so’ stories [64] that are based on subjective or coarse evidence. A sequence analysis would further refine our understanding of agonistic signaling in fight-bred lines. In addition to posturing, another novel aspect of our results are the guttural growls that were made by almost a quarter of the D Grade Category dogs in our study. These growls seem distinct from the acoustic properties of dog growls analyzed from multiple contexts [65] or other vocal descriptions in the literature [66,67]. It appears they may be a type of vocalization unique to the specific motivational state associated with extreme intraspecific aggression or to fight-bred lines, as they are not previously described in the literature. We are currently working on analyzing the guttural growl vocalizations with spectrographic analysis, so we can better compare specific acoustic properties against the published literature. If they are unique, they could serve as another diagnostic tool for identifying extreme intraspecific aggression.

Another key issue of theoretical and applied importance concerns the malleability of violent intraspecific aggression. Extensive data indicate that aggression becomes rewarding in the subset of individuals with violent phenotypes [68]. Indeed, another term used for impulsive aggression is ‘instrumental’ aggression, because the opportunity to attack a conspecific can be used as the unconditioned stimulus in an operant paradigm [69] and is likened to addiction [70]. Furthermore, while food manipulations and positive punishers can attenuate the display of violent aggression, the aggression is resistant to extinction and has a strong rebound upon cessation of differential reinforcement or punishment schedules [71]. Although this is not unique to violent aggression it is, perhaps, uniquely important as regards extreme aggressive behavior because of the dire consequences such aggression can have. Our study cannot speak to these features in differentially selected lines of domestic dogs. However, we raise this point because, given the degree of overlap between the rodent literature and fight-bred dogs in the quantitative characteristics of violent aggression, it is possible that resistance to extinction is also a shared feature of the phenotype across species. Resistance to extinction would pose serious risk and have profound implications for shelter staff determining outcomes for the *subset* of violent individuals in populations of fight-bred dogs.

There are limitations to our study design using a model. Because there was no direct interaction with a live conspecific, we lack definitive data that the dogs rated as extremely aggressive to the model in our sample would have exhibited the same aggression to a live conspecific. However, in our previous study we validated test dogs’ behavior to the model against their behavior to live conspecifics of both sexes in a large sample of hundreds of fight-bred dogs [38]. Our previous study clearly established the validity of the model dog as a tool to assess extreme intraspecific aggression in fight-bred lines and, therefore, offsets the lack of live conspecifics in this study. As explained in Reid and colleagues (2022), models allow expression of the consummatory phase of aggressive behavior in a way that is not possible to replicate with live test dogs and, thus, can be more informative in some cases. Because the behavior of severely aggressive dogs was the focal question of this study, our sample included more D Grade Category dogs to ensure capturing representative data for this group. We included a smaller comparator group primarily to ensure specific behaviors thought to be related to extreme aggression were not occurring in non-aggressive or ‘normally’ aggressive dogs, too. Therefore, comparisons between the D Grade Category and A:C Grade Category dogs should be interpreted cautiously, given the unequal sample sizes. Finally, there are several limitations to comparing our results for the regional targets of bites delivered to the model dog to published scar chart reports of injuries to living (or dead) canids. We reported the percentage of focal animals that bit the model in a specific region, whereas other papers report the percentage of recipients with damage to a specific region. The demarcations between body regions also vary between published studies, and they were necessarily less precise here, when coding bites from video vs documenting injuries or scars on live dogs and cadavers. This led us to combine some regions that were not easily distinguishable from video playback. Additionally, the model is a static recipient of bites, which is clearly different from a dyadic agonistic encounter between live conspecifics involving active defense of body regions [59].

## Conclusions

The broad and varied use of the term ‘aggression’ creates challenges in many disparate contexts: academic research attempting to understand its prevalence and/or risk factors, applied studies seeking to identify or validate behavior modification interventions, clinical cases that require assessment of treatment efficacy, criminal proceedings where liability must be determined, and in animal shelters that must make determinations of which animals are safe to place into the community and which should be euthanized. Despite the importance of nuanced and quantifiable distinctions, dogs are often classified in a binary manner as aggressive (or not) and discrete forms of aggression (to humans, conspecifics, etc.) are often lumped into a unitary ‘aggression’ scale.

The quantitative differences we report here underscore the usefulness and necessity of quantifying discrete behaviors when phenotyping animals. By utilizing established paradigms in a novel species, we found that extreme intraspecific aggression in fight-bred lines of domestic dogs shares a similar behavioral phenotype as impulsive aggression characterized in rodent models of violence. In both cases, directional selection for hypertrophied intraspecific aggression increases the incidence of impulsive or “pathological” aggression relative to the background population of non-selected lines. The characteristics of the aggression overlap, too, specifically: a significantly reduced latency to attack coupled with a significantly increased intensity of attack as measured by the frequency, duration, and target location of attacks. Such violent aggression is coupled with alterations in processing of or responsiveness to social cues – in rodents, evidenced by attacks to females or anesthetized conspecifics, in our study, by dramatic reductions in the prevalence and duration of investigatory behavior and integration of social information across cue modalities. Further, in both canid and rodent systems, experiential learning via fighting experience plays a role in the onset of such extreme aggression. Finally, and also in both cases, only a minority of individuals from directionally selected lines develop pathological aggression, regardless of experiential learning.

Our findings underscore the importance of assessing the behavior of dogs from fight-bred lines, to identify the many individuals who are *not* extremely aggressive despite their selection and life history. We also further validated the utility of models for detecting such aggression in fight-bred lines of domestic dogs, because the categorical ratings of experts were significantly related to quantitative metrics of aggression – thereby indicating the ratings are not the result of implicit or explicit rater biases, but rather accurately capture levels of aggressive behavior observed during the model subtest. Use of expert ratings is logistically more feasible when a large number of animals need to be evaluated and our findings support the use of ratings by behavior professionals with extensive training and experience, similar to findings for assessing working dog abilities. For scientific studies, however, we suggest a widespread adoption of standardized metrics for the frequency, latency, and intensity of physical attack. This will improve the rigor and interpretability of studies related to phenotyping aggression in the domestic dog.

## Supporting information

S1 Table The prevalence behaviors exhibited by severely aggressive dogs (Grade D, n = 92) and comparator subjects (Grades A, B, and C, n = 45 total, 15 per grade). The Number of Dogs column shows the tally of dogs exhibiting a given behavior within that Grade Category. The Percent column shows the tally of individuals expressed as a percentage of the grade sample. The Total Count column shows the sum of the number of bouts of a given behavior across all dogs in that sample. The Weighted Total was calculated as Total Count/ Number of Dogs to provide the average number of bouts for only those individuals expressing the behavior. The Total Duration column shows the sum of time (s) spent engaging in each behavior across all dogs in that Grade sample. The Average Percent of Test was calculated as the Total Duration/ Test Duration for each subject and behavior, with the resultant proportion of test duration averaged across dogs in each Grade Category and then multiplied by 100%. Together, the Percent of Grade Sample, Weighted Total and Average Duration as Proportion of Test provide information on the prevalence of behaviors across individuals with severe conspecific aggression (and the comparator groups), as well as the frequency and duration of expression within individuals.
(DOCX)
