# Supplementary material for: Topography of violent intraspecific aggression in a subset of dogs from directionally selected lines of Canis familiaris
Source: PLoS One. 2025 Nov 19;20(11):e0335278. doi: 10.1371/journal.pone.0335278 (PMC12629427; doi:10.1371/journal.pone.0335278)
Supplement: S1 Table — The Number of Dogs column shows the tally of dogs exhibiting a given behavior within that Grade Category. The Percent column shows the tally of individuals expressed as a percentage of the grade sample. The Total Count column shows the sum of the number of bouts of a given behavior across all dogs in that sample. The Weighted Total was calculated as Total Count/ Number of Dogs to provide the average number of bouts for only those individuals expressing the behavior. The Total Duration column shows the sum of time (s) spent engaging in each behavior across all dogs in that Grade sample. The Average Percent of Test was calculated as the Total Duration/ Test Duration for each subject and behavior, with the resultant proportion of test duration averaged across dogs in each Grade Category and then multiplied by 100%. Together, the Percent of Grade Sample, Weighted Total and Average Duration as Proportion of Test provide information on the prevalence of behaviors across individuals with severe conspecific aggression (and the comparator groups), as well as the frequency and duration of expression within individuals. (DOCX) [file pone.0335278.s001.docx]

**S1 Table.**

| **Behavior** | **Concern Severity Grade** | **Number of Dogs Exhibiting** | **Percent of Grade Sample** | **Total Count of Bouts** | **Weighted Total** | **Total Duration (seconds)** | **Average Percent of Test** |
| --- | --- | --- | --- | --- | --- | --- | --- |
| **Bite** | A | 1 | 6.7 | 1 | 1 | - | - |
|  | B | 0 | 0 | 0 | 0 | - | - |
|  | C | 4 | 26.7 | 41 | 10.25 | - | - |
|  | *All A-C* | *5* | *11.1* | *42* | *8.4* | *-* | *-* |
|  | D | 69 | 75 | 289 | 4.19 | - | - |
| ***Bite and Hold*** | A | 0 | 0 | 0 | 0 | 0 | 0 |
|  | B | 0 | 0 | 0 | 0 | 0 | 0 |
|  | C | 1 | 6.7 | 6 | 6 | 66 | 3.3 |
|  | *All A-C* | *1* | *2.2* | *6* | *6* | *66* | *1.1* |
|  | D | 49 | 53.3 | 98 | 2 | 1039 | 16.6 |
| **Bite and Shake** | A | 0 | 0 | 0 | 0 | - | - |
|  | B | 0 | 0 | 0 | 0 | - | - |
|  | C | 1 | 6.7 | 11 | 11 | - | - |
|  | *All A-C* | *1* | *2.2* | *11* | *11* | *-* | *-* |
|  | D | 39 | 42.4 | 173 | 4.44 | - | - |
| ***Biting, Fixated*** | A | 0 | 0 | 0 | 0 | 0 | 0 |
|  | B | 0 | 0 | 0 | 0 | 0 | 0 |
|  | C | 1 | 6.7 | 1 | 1 | 6 | 0.3 |
|  | *All A-C* | *1* | *2.2* | *1* | *1* | *6* | *0.1* |
|  | D | 32 | 34.8 | 46 | 1.44 | 559 | 9.1 |
| **Regrip** | A | 0 | 0 | 0 | 0 | - | - |
|  | B | 0 | 0 | 0 | 0 | - | - |
|  | C | 1 | 6.7 | 4 | 4 | - | - |
|  | *All A-C* | *1* | *2.2* | *4* | *4* | *-* | *-* |
|  | D | 26 | 28.3 | 129 | 4.96 | - | - |
| **Break Stick** | A | 0 | 0 | 0 | 0 | - | - |
|  | B | 0 | 0 | 0 | 0 | - | - |
|  | C | 0 | 0 | 0 | 0 | - | - |
|  | *All A-C* | *0* | *0* | *0* | *0* | *-* | *-* |
|  | D | 6 | 6.5 | 8 | 1.33 | - | - |
| **Growl** | A | 0 | 0 | 0 | 0 | - | - |
|  | B | 0 | 0 | 0 | 0 | - | - |
|  | C | 1 | 6.7 | 1 | 1 | - | - |
|  | *All A-C* | *1* | *2.2* | *1* | *1* | *-* | *-* |
|  | D | 10 | 10.9 | 17 | 1.7 | - | - |
| **Guttural Growl** | A | 0 | 0 | 0 | 0 | - | - |
|  | B | 0 | 0 | 0 | 0 | - | - |
|  | C | 0 | 0 | 0 | 0 | - | - |
|  | *All A-C* | *0* | *0* | *0* | *0* | *-* | *-* |
|  | D | 21 | 22.8 | 38 | 1.8 | - | - |
| **Knockdown** | A | 4 | 26.7 | 7 | 1.75 | - | - |
|  | B | 3 | 20 | 6 | 2 | - | - |
|  | C | 12 | 80 | 26 | 2.17 | - | - |
|  | *All A-C* | *19* | *42.2* | *39* | *2.05* | *-* | *-* |
|  | D | 81 | 88 | 126 | 1.56 | - | - |
| ***Pin*** | A | 3 | 20 | 6 | 2 | 39 | 3.0 |
|  | B | 1 | 6.7 | 2 | 2 | 8 | 0.7 |
|  | C | 3 | 20 | 3 | 1 | 51 | 4.7 |
|  | *All A-C* | *7* | *15.6* | *11* | *1.57* | *98* | *2.8* |
|  | D | 54 | 58.7 | 83 | 1.54 | 776 | 11.7 |
| ***Clasp & Thrust*** | A | 2 | 13.3 | 3 | 1.5 | 20 | 1.8 |
|  | B | 3 | 20 | 4 | 1.33 | 39 | 3.1 |
|  | C | 8 | 53.3 | 16 | 2 | 127 | 11.5 |
|  | *All A-C* | *13* | *28.9* | *23* | *1.77* | *186* | *5.1* |
|  | D | 13 | 14.1 | 22 | 1.69 | 213 | 2.9 |
| ***Clasp*** | A | 2 | 13.3 | 3 | 1.5 | 5 | 0.4 |
|  | B | 1 | 6.7 | 1 | 1 | 7 | 0.5 |
|  | C | 5 | 33.3 | 7 | 1.4 | 27 | 2.3 |
|  | *All A-C* | *8* | *17.8* | *11* | *1.38* | *39* | *1.1* |
|  | D | 31 | 33.7 | 42 | 1.35 | 237 | 5.3 |
| ***Clasping, Fixated*** | A | 0 | 0 | 0 | 0 | 0 | 0 |
|  | B | 0 | 0 | 0 | 0 | 0 | 0 |
|  | C | 1 | 6.7 | 1 | 1 | 3 | 0.2 |
|  | *All A-C* | *1* | *2.2* | *1* | *1* | *3* | *0.07* |
|  | D | 21 | 22.8 | 24 | 1.14 | 105 | 2.1 |
| ***Ride Up*** | A | 7 | 46.7 | 20 | 2.86 | 36 | 3.5 |
|  | B | 5 | 33.3 | 11 | 2.2 | 53 | 3.9 |
|  | C | 12 | 80 | 24 | 2 | 91 | 8.3 |
|  | *All A-C* | *24* | *53.3* | *55* | *2.29* | *180* | *5.2* |
|  | D | 67 | 72.8 | 111 | 1.66 | 346 | 5.5 |
| ***Stand Over*** | A | 2 | 13.3 | 2 | 1 | 15 | 1.0 |
|  | B | 0 | 0 | 0 | 0 | 0 | 0 |
|  | C | 4 | 26.7 | 7 | 1.75 | 36 | 3.6 |
|  | *All A-C* | *6* | *13.3* | *9* | *1.5* | *51* | *1.5* |
|  | D | 13 | 14.1 | 15 | 1.15 | 64 | 0.8 |
| ***Stare*** | A | 2 | 13.3 | 2 | 1 | 4 | 0.6 |
|  | B | 0 | 0 | 0 | 0 | 0 | 0 |
|  | C | 0 | 0 | 0 | 0 | 0 | 0 |
|  | *All A-C* | *2* | *4.4* | *2* | *1* | *4* | *0.5* |
|  | D | 4 | 4.3 | 9 | 2.25 | 38 | 0.5 |
| ***T Position Dominant*** | A | 0 | 0 | 0 | 0 | 0 | 0 |
|  | B | 0 | 0 | 0 | 0 | 0 | 0 |
|  | C | 0 | 0 | 0 | 0 | 0 | 0 |
|  | *All A-C* | *0* | *0* | *0* | *0* | *0* | *0* |
|  | D | 5 | 5.4 | 5 | 1 | 27 | 0.7 |
| ***Tail Flag*** | A | 0 | 0 | 0 | 0 | 0 | 0 |
|  | B | 0 | 0 | 0 | 0 | 0 | 0 |
|  | C | 1 | 6.7 | 1 | 1 | 8 | 0.9 |
|  | *All A-C* | *1* | *2.2* | *1* | *1* | *8* | *0.3* |
|  | D | 1 | 1.1 | 1 | 1 | 2 | 0.04 |
| ***Head Over*** | A | 0 | 0 | 0 | 0 | 0 | 0 |
|  | B | 2 | 13.3 | 2 | 1 | 2 | 0.2 |
|  | C | 2 | 13.3 | 2 | 1 | 6 | 0.5 |
|  | *All A-C* | *4* | *8.9* | *4* | *1* | *8* | *0.23* |
|  | D | 5 | 5.4 | 5 | 1 | 6 | 0.09 |
| **Avoid** | A | 6 | 40 | 8 | 1.3 | 43 | 5.9 |
|  | B | 5 | 33.3 | 8 | 1.6 | 27 | 3.2 |
|  | C | 0 | 0 | 0 | 0 | 0 | 0 |
|  | *All A-C* | *11* | *24.4* | *16* | *1.45* | *70* | *3* |
|  | D | 0 | 0 | 0 | 0 | 0 | 0 |
| ***Inspect*** | A | 12 | 80 | 22 | 1.8 | 74 | 9.3 |
|  | B | 14 | 93.3 | 35 | 2.5 | 107 | 9.4 |
|  | C | 11 | 73.3 | 23 | 2.1 | 89 | 8.3 |
|  | *All A-C* | *37* | *82.2* | *80* | *2.16* | *270* | *9* |
|  | D | 36 | 39.1 | 53 | 1.47 | 176 | 2.5 |
| ***Sniff*** | A | 15 | 100 | 50 | 3.33 | 245 | 27.8 |
|  | B | 15 | 100 | 57 | 3.8 | 303 | 31.2 |
|  | C | 11 | 73.3 | 33 | 3 | 170 | 16.2 |
|  | *All A-C* | *41* | *91.1* | *140* | *3.4* | *718* | *25.1* |
|  | D | 68 | 73.9 | 160 | 2.35 | 754 | 11.2 |
